# Assessing vocal performance in complex birdsong: a novel approach

**DOI:** 10.1186/s12915-014-0058-4

**Published:** 2014-08-06

**Authors:** Nicole Geberzahn, Thierry Aubin

**Affiliations:** Centre National de la Recherche Scientifique, Centre de Neuroscience Paris Sud, UMR 8195, 91405 Orsay, France; Université Paris Sud, Equipe Communications Acoustiques/CNPS, Bat. 446, 91405 Orsay, France; Current address: Laboratoire Éthologie Cognition Développement, Université Paris Ouest Nanterre La Défense, 200 Avenue de la République 92001, Nanterre Cedex, France

**Keywords:** Aggressive signaling, *Alauda arvensis*, Birdsong, Complex song, Contextual variation, Production limits

## Abstract

**Background:**

Vocal performance refers to the ability to produce vocal signals close to physical limits. Such motor skills can be used by conspecifics to assess a signaler’s competitive potential. For example it is difficult for birds to produce repeated syllables both rapidly and with a broad frequency bandwidth. Deviation from an upper-bound regression of frequency bandwidth on trill rate has been widely used to assess vocal performance. This approach is, however, only applicable to simple trilled songs, and even then may be affected by differences in syllable complexity.

**Results:**

Using skylarks (*Alauda arvensis*) as a birdsong model with a very complex song structure, we detected another performance trade-off: minimum gap duration between syllables was longer when the frequency ratio between the end of one syllable and the start of the next syllable (inter-syllable frequency shift) was large. This allowed us to apply a novel measure of vocal performance - vocal gap deviation: the deviation from a lower-bound regression of gap duration on inter-syllable frequency shift. We show that skylarks increase vocal performance in an aggressive context suggesting that this trait might serve as a signal for competitive potential.

**Conclusions:**

We suggest using vocal gap deviation in future studies to assess vocal performance in songbird species with complex structure.

**Electronic supplementary material:**

The online version of this article (doi:10.1186/s12915-014-0058-4) contains supplementary material, which is available to authorized users.

## Background

Displays used in animal communication are often subject to physical constraints, such as biomechanical limits arising during their production, and only highly skilled individuals should be capable of performing challenging displays. Consequently, display traits linked to production limits may indicate a signaler’s quality or motivational state in a variety of contexts such as mate choice, male-male competition, or during predator-prey interaction. Examples are prey animals performing displays of agility or speed indicating their ability to escape [1,2], bird species performing acrobatic aerial displays to attract females [3], or male songbirds using their vocal skills in male-male vocal interactions or female attraction [4].

Birdsong is a fruitful model for studying how performance limits affect signal properties: vocal production requires precise central nervous control of respiration, modulation of the sound-producing organ, the syrinx, and vocal tract modulation [5,6]. Different traits of birdsong could be subject to performance limits, such as the consistency in performing recurring vocal elements, termed syllables [7]; song density - the proportion of sound per unit of time [8]; low-pitched vocalizations [9]; or song amplitude [10,11]. One song trait that has received particular attention is trill performance (reviewed in [4,12,13]). In trilled song components, a given syllable type is repeated several times. In such components, trill rate might be limited by itself and in addition, evidence from several species suggests that there is a structural trade-off between the frequency bandwidth and the rate at which syllables are repeated (reviewed in [4]). A candidate mechanism for this trade-off is the vocal tract, which has to be tuned to the fundamental frequency produced by the syrinx in order to suppress energy in higher harmonics; the movements required for tuning the vocal tract are constrained by the speed of the involved motor systems [5,14]. Additionally, the frequency control mechanism in the syrinx itself could be subject to speed limits although birds might overcome such limits at the sound source by switching between the different sides of the syrinx (for example, see [15]). Finally, respiratory requirements certainly play a role as well, because the duration of silent gaps between syllables is likely to be regulated by respiratory needs. Canaries (*Serinus canaria*), for instance, use a mini-breath respiratory pattern during song production in which volumes of air expired during phonation are closely matched to those inspired during silent gaps [16]. Thus, respiratory requirements may also limit an increase in trill rate.

Podos *et al*. [4] suggested that performance difficulty may vary with the structure of trills: If trills consist for instance mainly of down-sweeping syllables (or mainly of up-sweeping syllables), a subsequent syllable will often begin at a different frequency as the previous syllable ended (for example, banded wren, *Thryothorus pleurostictus* [17]; swamp sparrows, *Melospiza georgiana* [18]; compare with figure one B in [4]). Accordingly, the vocal tract will need to be largely reconfigured during the silent gaps between two consecutive syllables. This might strongly limit the trill rate as such reconfiguration needs some time. Contrarily, in songbird species singing trills mainly consisting of an alternating sequence of down- and up-sweeping syllables, a syllable might begin at the same frequency as the preceding one ended (for example, house wrens, *Troglodytes aedon* [19,20]; dark-eyed junco, *Junco hyemalis* [21]; compare with figure one A in [4]). Thus, males may be able to shorten gaps between such syllables more easily as the vocal tract does not need to be largely reconfigured. This may allow them to increase the rate of syllable production more easily. In addition to such variation with the structure of trills, performance difficulty might also be affected by other parameters such as frequency or amplitude modulations within syllables or the degree of tonality.

**Figure 1:**
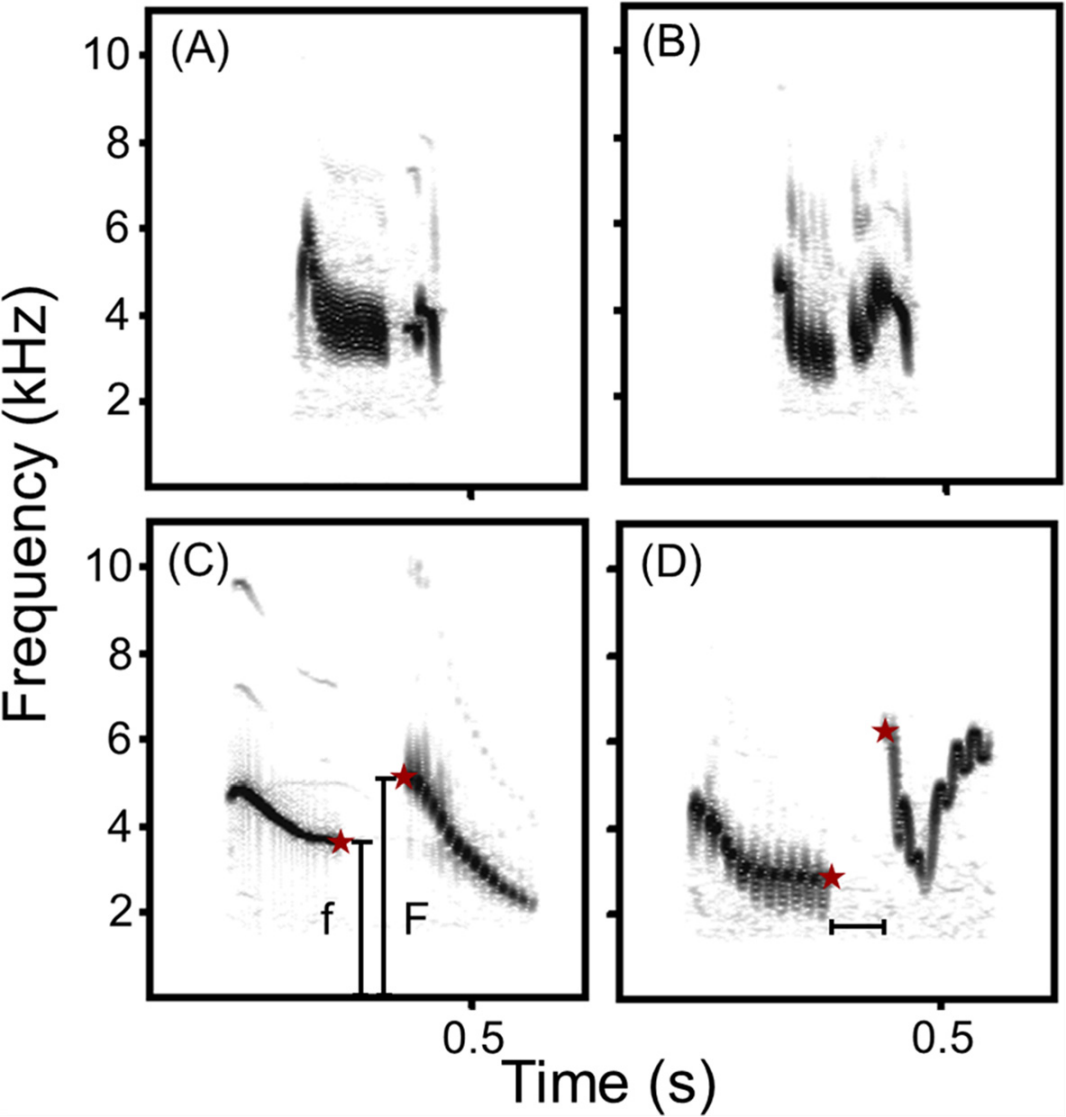
**Spectrograms of subsequent syllables produced by a male skylark in response to a territorial playback. (A,B)** Examples with small gaps. **(C,D)** Examples with large gaps. Red stars indicate peak frequency of the end of the first syllable and the start of the subsequent syllable. Inter-syllable frequency ratio was calculated by dividing the larger peak frequency (vertical bar ‘F’) by the smaller one (vertical bar ‘f’). The horizontal bar indicates gap duration.

Assessing vocal performance in songbird species with a complex song thus requires novel measures that are independent of such differences. We here applied such a measure, which we call ‘vocal gap deviation’, to assess modulation of vocal performance in an aggressive context in skylarks, a songbird with an extremely complex and versatile song. Vocal gap deviation is based on the assumption that the gap duration between two successive syllables is traded off against the inter-syllable frequency shift (assessed as ratio of frequency from the end of one syllable to the start of the next syllable, Figure 1C). This should impede the production of syllable transitions with both large inter-syllable frequency shifts and very short gaps between syllables. Vocal gap deviation assesses how closely a male is performing at this limit and is thus related to ‘vocal deviation’, which assesses the speed of frequency modulation in a trill [22]. Vocal gap deviation is, however, more broadly applicable as it is not restricted to a given succession of trills and allows estimation of vocal performance in a species with a complex song structure.

In the current study, we reanalyzed a dataset published earlier [8] in which skylarks sang in response to a territorial playback (reactive singing) and in baseline condition (spontaneous singing). We measured gap duration and related it to inter-syllable frequency shift in corresponding syllables. Our first hypothesis was that gap duration is traded off against inter-syllable frequency shift in the song of skylarks. Because males should be easily capable of singing with short gaps when inter-syllable frequency shifts are small but should require longer gaps to produce large inter-syllable frequency shifts, we expected to find a triangular distribution of data points when plotting these two parameters against each other with a significant lower-bound regression. Constraints are often reflected in such triangular distributions, because data falls only on one side of a boundary [23]. An alternative explanation for a trade-off between gap duration and inter-syllable frequency shifts could be that larger inter-syllable frequency shifts are associated with longer syllables (because longer syllables could have more time to modulate to extreme frequencies). In this case, longer gaps could simply be a consequence of larger respiration requirements rather than motor constraints caused by vocal tract reconfiguration. To assess this possibility, we tested for a relationship between syllable duration and inter-syllable frequency shifting.

We recently reported [8] that skylarks increase the sound density of their song, as compared to baseline singing, in an aggressive context and they seem to do so by switching to a different set of syllables for which the subsequent gap durations are smaller. This new and context-specific set of syllables might be associated with smaller inter-syllable frequency shifts allowing for smaller gap durations. Therefore, our second hypothesis was that skylarks decrease gap durations in reactive singing by producing unique, context-specific syllables that have syllable transitions with smaller inter-syllable frequency shifts compared to the context-specific syllables produced uniquely in spontaneous singing. We predicted that inter-syllable frequency shifts would be significantly smaller in syllables performed in response to the territorial challenge and in particularly in those that were unique to this context. Third, we hypothesized that skylarks sing closer to the performance limit when challenged by a territorial playback. If this is the case, the deviation from a lower-bound regression should be smaller in reactive than in spontaneous singing.

## Results

### Relationship between gap duration and inter-syllable frequency shift

**Figure 2:**
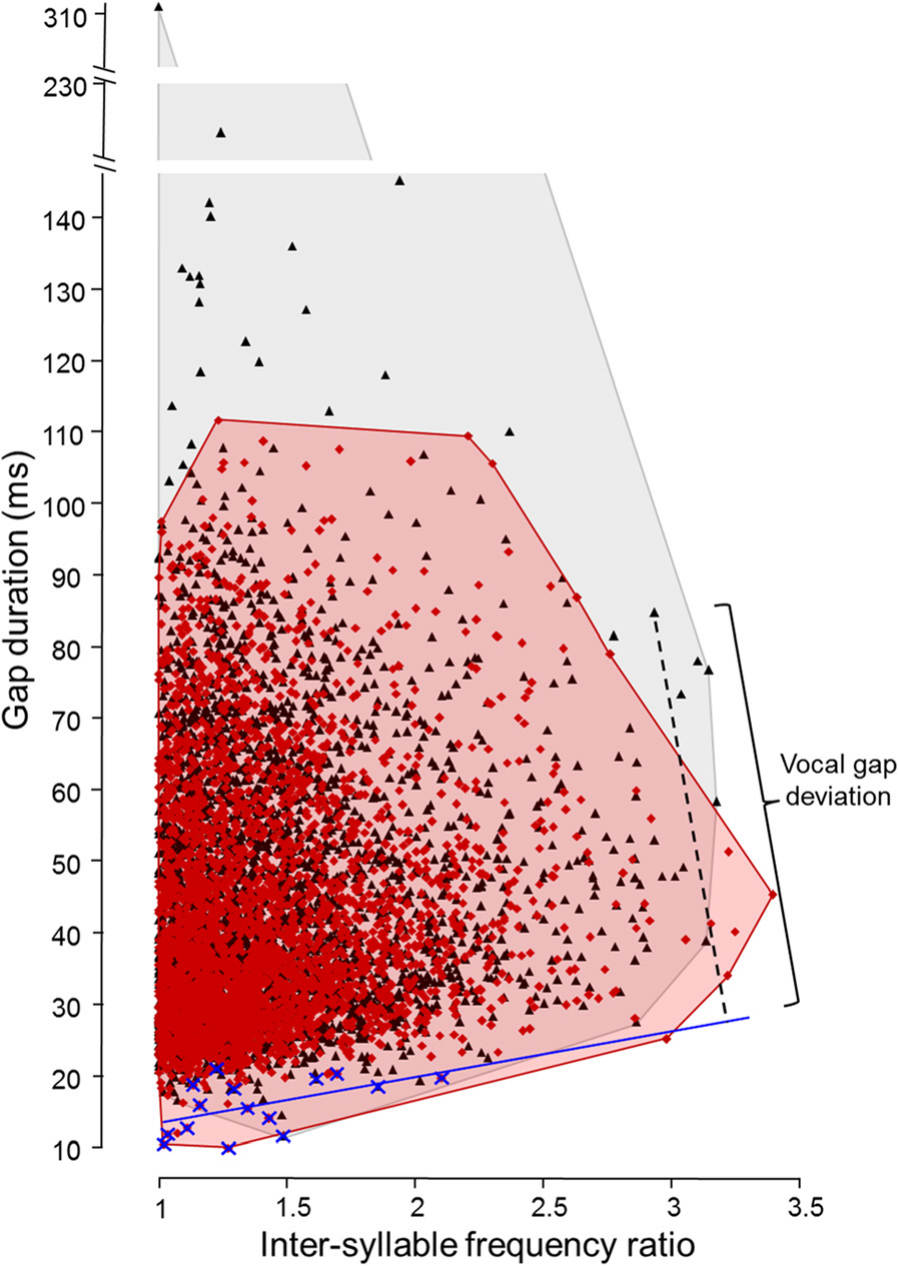
**Gap duration plotted against inter-syllable frequency ratio.** Blue line: lower-bound regression line. Red diamonds: syllables produced in response to territorial playbacks. Black triangles: syllables produced in spontaneous song (both contexts together n = 6,910 syllables of 16 subjects). Blue crosses: data points used to calculate the lower-bound regression line assessed by the equal samples per bin method [40]. Vocal gap deviation is measured as the minimum orthogonal distance of each data point to the lower-bound regression line; an example is shown for one data point (black dotted line). Minimum area convex polygon is given in red for reactive and in grey for spontaneous singing. For statistics see text.

When plotting gap duration against inter-syllable frequency ratio for all syllables measured, we revealed a triangular distribution (Figure 2). The lower-bound regression, which represents the putative performance limit, had a significant positive slope (y = 6.3963x + 6.9766; linear regression: F_1,13_ = 4.93, r^2^ = 0.27, *P* = 0.045; Figure 2).

### Longer syllables are not associated with larger inter-syllable frequency shifts

Longer syllables could be associated with higher inter-syllable frequency ratios as they have more time to modulate to extreme frequencies. Thus, longer gaps following higher inter-syllable frequency ratios could simply be a consequence of larger respiration requirements to replenish air exhaled during long syllables. However, our data did not reveal such relationships between syllable duration and inter-syllable frequency ratios: only 12 out of 32 subject-wise correlation tests had positive coefficients, only one of which was significant (corresponding to the expected proportion of false positive findings, Table 1).

**Table 1:** **No relationship between syllable duration and inter-syllable frequency ratios**

**Subject**	**Spontaneous singing**		**Reactive singing**	
	**rho**	***P***	**rho**	***P***
1	-0.08	0.35	-0.16	**0.04**
2	-0.06	0.52	-0.14	0.10
3	0.07	0.60	0.15	0.09
4	-0.10	0.32	-0.10	0.29
5	0.20	**0.01**	0.09	0.18
6	-0.02	0.82	-0.14	0.13
7	-0.01	0.93	0.01	0.91
8	-0.17	0.11	-0.05	0.56
9	-0.13	0.25	0.06	0.54
10	-0.09	0.33	-0.19	0.08
11	-0.001	0.99	0.08	0.42
12	0.03	0.70	0.14	0.16
13	-0.08	0.51	0.003	0.98
14	0.12	0.20	0.10	0.35
15	-0.06	0.56	-0.04	0.63
16	-0.11	0.24	-0.15	0.10

Spearman’s rank rho is given for correlations between syllable duration and inter-syllable frequency ratios of each of the 16 subjects in spontaneous and reactive singing. Tests are based on mean values per syllable type. Significant *P*-values are given in bold.

### Inter-syllable frequency shift alone does not vary with the context

Skylarks did not shift the frequency between syllables to a lesser degree in reactive than in spontaneous singing: we could not detect a significant contextual difference in the inter-syllable frequency ratio when comparing all syllables (spontaneous singing: mean ± standard deviation (SD) 1.43 ± 0.04; reactive singing: 1.42 ± 0.04; paired t-test, t = -0.85, degrees of freedom (df) = 15, *P* = 0.41) nor when considering only context-specific syllable types (spontaneous singing: mean ± SD 1.43 ± 0.04; reactive singing: 1.42 ± 0.05; paired t-test, t = -0.26, df = 15, *P* = 0.8).

### Skylarks sing closer to the performance limit when challenged by a territorial playback

**Figure 3:**
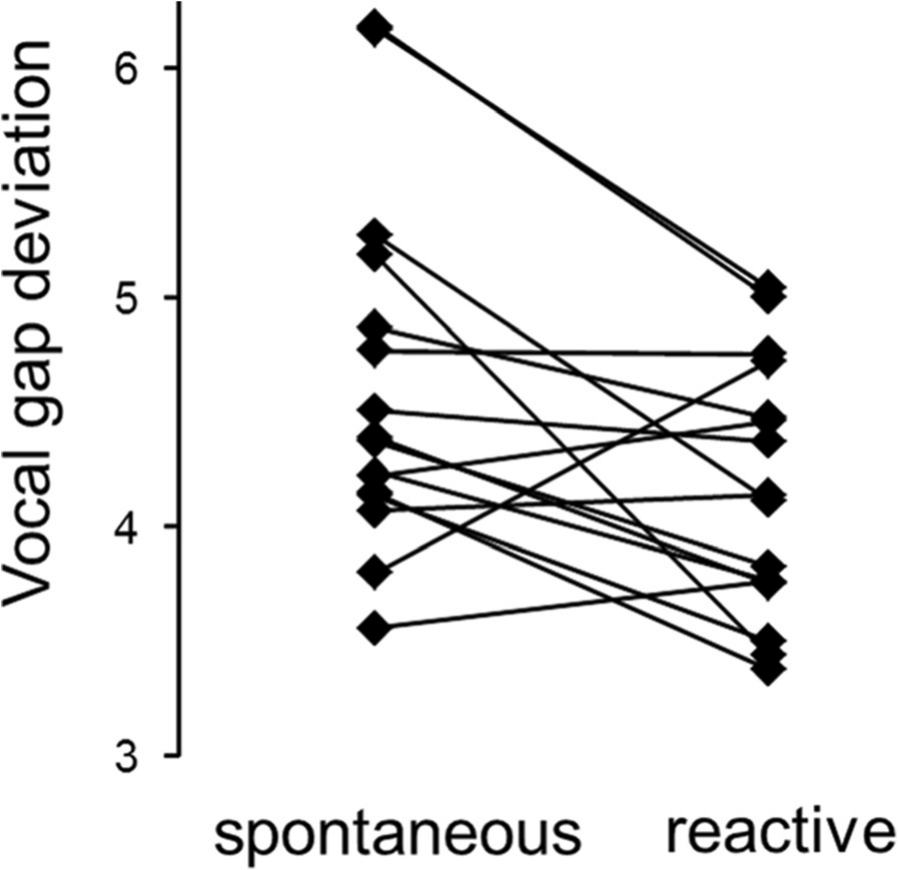
**Vocal gap deviation was smaller when skylarks were singing in response to a territorial playback.** This indicates that they were singing closer to their performance limit when challenged than when singing spontaneously. Average values of all syllable types are shown for each of 16 subjects. For statistics see text.

Visual examination of the distribution of all syllables in Figure 2, in which the gap durations are plotted against the inter-syllable frequency ratios, revealed that some of the syllables sung in the baseline condition (spontaneous song) were far above the lower-bound regression line, suggesting that males were singing far below their performance capacities as far as the trade-off between inter-syllable frequency shift and gap duration is concerned. At the same time, some of the syllables produced in reactive song seemed to be closer to the lower-bound regression line. In fact, when taking into account all syllables, vocal gap deviation was significantly smaller when birds were singing in response to the territorial playback than when singing spontaneously (spontaneous singing: mean ± SD 4.61 ± 0.76; reactive singing: 4.15 ± 0.55; paired t-test, t = -2.76, df = 15, *P* = 0.01; Figure 3).

To understand how skylarks arrive at singing closer to this performance limit, we examined contextual variation in two subsets of syllable types: context-specific syllable types and context-independent syllable types (the later termed ‘shared syllables’ in [8]). Vocal gap deviation was significantly smaller when considering only context-specific syllable types (spontaneous singing: mean ± SD 4.68 ± 0.8; reactive singing: 4.2 ± 0.65; paired t-test, t = -2.21, df = 15, *P* = 0.04). We observed the same trend for context-independent syllable types (spontaneous singing: mean ± SD, 4.54 ± 0.93; reactive singing: 4.24 ± 1.01; Wilcoxon signed rank test, V = 33, *P* = 0.07).

## Discussion

We found a triangular relationship between gap duration and inter-syllable frequency shift with a significant lower-bound regression in the song of skylarks. This relationship cannot be explained by a correlation of inter-syllable frequency shift with syllable duration, which might lead to longer gap durations due to respiratory requirements. Thus, gap duration seems to be traded-off against inter-syllable frequency shift, which suggests a performance limit that skylarks face during song production. Contrary to our expectation, inter-syllable frequency shift was not significantly smaller when males were singing in response to a playback of conspecific song simulating a territorial intrusion. However, vocal gap deviation (the orthogonal distance of data points to the lower-bound regression) was significantly smaller when singing reactively than when singing spontaneously, indicating that skylarks performed closer to the performance limit when challenged.

### Structure-dependent variation of vocal performance

The observation that gap duration is related to inter-syllable frequency shift in the complex song of skylarks supports the hypothesis that it takes males more time to pass from one syllable to the next if the change in frequency between the end of the preceding syllable and the start of the subsequent syllable is large. Likewise, it seems to be easier for them to sing with shorter gaps if the inter-syllable frequency ratio is small. A candidate mechanism proposed for this trade-off is the temporal requirements of the motor system to tune the vocal tract to the fundamental frequency in order to suppress energy in higher harmonics [5,14]. This interpretation is supported by the pure-tone quality of skylark song. The necessity to replenish air supply and thus to ensure oxygen delivery certainly also constraints the brevity of silent gaps in between syllables in the skylark, a continuous singer that sings during flight. In this species, gap duration correlates with preceding syllable duration [24], suggesting that skylarks exhale more air when producing longer syllables and thus require longer gaps for inhaling. We could not, however, find an association between inter-syllable frequency ratios and syllable durations. We can therefore rule out that the reported trade-off (that is, between gap duration and inter-syllable frequency ratio) arises simply because syllables with larger frequency shifts are the ones with longer syllable durations that need longer subsequent gaps.

Our findings are consistent with the idea that performance difficulties vary with the structure - a hypothesis originally proposed by Podos *et al*. [4]. These authors suggested that the production of syllables in which the end of one syllable is produced at a different frequency than the start of the subsequent syllable might be more likely to be subject to performance constraints than syllables in which the inter-syllable frequency shift is small. Indeed, studies on species with trilled song components containing mainly either up- or down-sweeps have found a relationship between vocal performance and the aggressive motivation or quality of males (for example, swamp sparrows [25], banded wren, *Thryophilus pleurostictus* [26]) whereas most studies on species with trill components also made up by chevron-shaped syllables or consisting of an alternating sequence of down- and up-sweeping syllables so far failed to find such relationships (for example, house wren [19,20], dark-eyed junco [21], but see [27]). We here provide clear empirical evidence supporting the hypothesis that performance constraints vary with the structure of syllable sequences of birdsong.

### No context-dependent modulation of inter-syllable frequency shift

In a previous study, we found that gaps following those syllables that skylarks produced uniquely in an aggressive context were shorter than for syllables produced uniquely in baseline condition [8]. This suggests that skylarks might selectively choose such syllable transitions in which inter-syllable frequency shifting is small in order to allow for small gap durations and consequently higher sound density. However, this hypothesis is not supported by our data as we failed to detect contextual modulation of inter-syllable frequency shift. Actually, skylarks may be limited in the freedom to recombine syllables. This might be due to, for example, the function of sequential organization in neighbor-stranger discrimination in this species [28]: more than 75% of the song is organized in recurring sequences of syllables called phrases. Phrases that neighbors have in common with each other convey information on group membership and allow for reduced aggression between established neighbors [28,29]. On a more proximate level, recombination of syllables might be restricted by the song learning process as sequential information might be included in this process as already shown for nightingales (*Luscinia megarhynchos* [30,31]).

### Skylarks sing closer to their performance limit when challenged

Vocal gap deviation was smaller when skylarks were singing in response to a territorial playback than when singing in baseline condition. Thus, males performed closer to the vocal production limit, which seemed to keep them from increasing the inter-syllable frequency shift and decreasing gap duration at the same time. When considering only context-specific syllable types we cannot rule out that this finding simply reflects the fact that gap duration alone varies with the context. We have previously shown that syllable types produced uniquely in reactive singing are followed by smaller gaps than those produced uniquely in spontaneous singing [8]. Perhaps the difference in vocal gap deviation is mainly driven by this difference in gap duration. However, the picture changes when considering all syllable types: here the newly introduced measure of vocal performance allowed us to capture contextual variation that could not be observed in the two parameters going into its computation: we could neither detect contextual variation in gap duration alone [8] nor in inter-syllable frequency shifting alone (this study). Vocal gap deviation, thus, provides a better biological explanation of the overall variation observed in the song of skylarks.

Singing close to a performance limit has been well documented for different songbird species in the context of female attraction (for example, canaries [32]) and male-male competition (for example, swamp sparrows [25]). Studies on species with trilled song structure document a relationship between high vocal performance and male quality, condition, age, or aggressive motivation (for example [26,33]). In skylarks, contextual variation in vocal gap deviation was most pronounced when analyzing all syllable types together; differences were less pronounced (but still significant) for context-specific syllable types alone and became marginally non-significant for context-independent syllable types. Thus, we cannot draw conclusions on which type of syllables might drive the differences, and further studies will be needed to understand how skylarks arrive at lower vocal gap deviations during reactive singing. In addition, vocal gap deviation might be just one of multiple different song features that can indicate male skylarks’ aggressive motivation and/or quality. Thus, we cannot rule out that syllables performed below the capacities concerning the trade-off between gap duration and inter-syllable frequency shift might nevertheless indicate the competitive potential of a male in terms of other acoustic features.

Searcy and Beecher [34] postulated three criteria that should be met to establish that a given singing behavior is an aggressive signal. The signal value should increase in aggressive contexts (context criterion). The signal should predict aggressive escalation by the signaler (predictive criterion). And differential signal values should elicit differential responses (response criterion). In the current study, we showed that vocal performance - measured as vocal gap deviation - meets the context criterion and might therefore give a signal for competitive potential in skylarks. It would be very interesting to test in the future whether the predictive and the response criterion are met as well in skylarks, and whether vocal gap deviation is related to quality, condition, reproductive success, or age.

## Conclusion

We introduced a new measure of vocal performance, the ‘vocal gap deviation’. This measure is based on the same assumption as the widely used ‘vocal deviation’, namely that modulating the configuration of the vocal apparatus in order to track changes in the fundamental frequency requires a minimum amount of time. It assumes that both the production of broadband trills with very high trill rate as well as large inter-syllable frequency shifts with very short gap durations should be physically challenging. The advantage of vocal gap deviation as a measure is that it can be applied to non-trilled components of song and to birdsong with a very complex structure. In addition, this measure can assess vocal performance independent of variation in other song features such as frequency or amplitude modulations within syllables or the degree of tonality. Using this measure on skylarks, we showed that males decreased vocal gap deviation and thus increased vocal performance when being challenged by a simulated territorial intrusion, suggesting that this trait might serve as a signal for aggressive motivation in skylarks.

## Methods

### Study site, subjects and their song

The study was conducted on sixteen male skylarks at nine different locations in the agricultural fields surrounding the University of Paris 11, France, during the 2011 (N = 9 subjects, 9 May to 1 July) and 2012 (N = 7 subjects, 3 May to 20 June) breeding seasons. Birds were not individually marked as we did not succeed in catching them. However, we are confident that we were able to identify individual subjects by carefully observing position and behavior, especially the conspicuous flight song, repeatedly displayed at a given location. Furthermore, site fidelity is very strong in breeding skylarks [35] and boundaries between adjoining territories are stable once territories are established [36]. Skylarks have a large repertoire of different syllable types (>300 [28]) and perform their syllables in a continuous fashion with short inter-syllable gaps. Furthermore, song is predominantly produced during flight. Songs produced during flight last on average for 261 s [37] but can last up to 1 h (NG, personal observation). Syllables are either sung with ‘immediate variety’, males switching to a new syllable type with each syllable produced, or with ‘eventual variety’, a given syllable type being repeated several times to constitute trilled components of the song.

### Recording methods and playback experiments

Song recordings were made at a sample rate of 44.1 kHz using a Sennheiser ME62/K6 omnidirectional microphone (frequency response: 20 Hz to 20 kHz ± 1 dB) mounted on a Telinga Universal parabola (diameter: 50 cm, Telinga, Tobo, Sweden) and connected to a Marantz PMD 670 solid-state recorder (Marantz, Kanagawa, Japan). We recorded songs between 0800 and 1300 hours. Spontaneous singing was defined as singing without any indication of interactions with a conspecific. We conducted playback experiments to simulate a territorial intrusion. Such experiments elicited territorial responses consisting of singing behavior, as well as approaching the simulated intruder, landing in its vicinity, or flying low over it. We recorded the song produced by the tested subject during the playback and in the 10 min after the end of the presentation of the playback stimulus (for further details see [8]).

### Song analysis

In the current paper we reanalyzed a dataset published earlier [8] using Avisoft SASLAB Pro. For this dataset we had previously selected one song recording with the highest signal-to-noise ratio for each context and each subject, and we had analyzed the first 40 s of the song corresponding approximately to the ascending phase of the flight [38]. Songs had been high-pass filtered (cut-off frequency: 1.4 kHz). For the current study, we created spectrograms to measure peak frequency at the end and the start of syllables (Fast Fourier transformation (FFT) length 1,024; frame 100%; overlap 75%, Hamming window, frequency resolution 43 Hz, temporal resolution of 5.8 ms) using the Automatic Parameter Measurements setup based on manual delineations of on- and offsets of syllables in oscillograms. Likewise, we measured gap and syllable durations from the same delineations directly on oscillograms. We defined a syllable as a continuous trace on the sound spectrogram or a group of continuous traces spaced out by less than 25 ms in all their renditions. Classification of syllables into syllable types was based on visual comparison of overall frequency modulation shapes in spectrograms by one observer (a subset of 947 syllables was controlled by a second observer who agreed in 94% of classifications).

We assessed ‘inter-syllable frequency shift’ as the ratio between the end peak frequency of a given syllable and the start peak frequency of the subsequent syllable. We used frequency ratios because linear frequency differences could give biased results by overestimating changes in the higher- relative to lower-frequency samples (compare with [39]). To this end, we divided the peak frequency with the higher value (either the end of the first or the start of the subsequent syllable) by the smaller peak frequency such that the minimum ratio was equal to 1 in the case where both peak frequencies adopted the same value. We then related gap duration between syllables to inter-syllable frequency ratio (Figure 1). To assess whether gap duration is traded-off against inter-syllable frequency ratio, we conducted a lower-bound regression analysis following methods established by Podos *et al*. [22]. To avoid sampling biases, we binned measurements of inter-syllable ratio according to the equal samples per bin method [40]. We used a modified version of the traditional upper-bound regression method established by Podos *et al*. [22]: we calculated frequency ratios instead of linear frequency differences, used the equal samples per bin method rather than equally sized bins, and plotted the temporal parameter against the frequency parameter rather than the reverse and thus used lower- instead of upper-bound regression. The later was done as inter-syllable frequency ratio is more likely to limit gap duration than the reverse. Using the traditional upper-bound regression method (on a plot of linear frequency differences against gap duration) instead of our modified approach did not change the results (see Additional files 1, 2 and 3).

To examine whether smaller gap durations in reactive singing were related to a lesser degree in inter-syllable frequency shifting, we compared the inter-syllable frequency ratios in all syllables and in context-specific syllables (termed ‘unshared syllables’ in [8]) between the spontaneous and reactive song. Context-specific syllables are unique to one of the contexts, that is, they can be found only either in spontaneous song (and not in reactive song) or only in reactive song (but not in spontaneous song). When comparing all syllables, we considered those context-specific syllables as well as those syllables that occurred both in spontaneous and in reactive song. To examine whether skylarks sing closer to a performance limit when challenged by a territorial playback, we calculated the ‘vocal gap deviation’ of all syllables as the minimum orthogonal distance to the lower-bound regression line (Figure 2). Note that due to properties of similar triangles, the horizontal and vertical distance of a given syllable to the lower-bound regression line are both proportional, and then correlated, to the orthogonal distance. Thus, analyzing horizontal and vertical instead of the orthogonal distance would give the same results.

Values of vocal gap deviation that fell below the lower-bound regression line were assigned a negative deviation (compare [20,41]). Greater values of vocal gap deviation represent lower performance and smaller (or negative) values of deviation reflect higher performance. When comparing parameters (inter-syllable frequency ratio, vocal gap deviation) between spontaneous and reactive singing, we calculated the average value for each subject based on average values for each given syllable type that a male produced.

### Statistical analysis

Statistical analyses were conducted using R2.13.0 [42]. We tested for normality using Shapiro-Wilk tests. In cases of normal distributions, we used a paired t-test to compare parameters in spontaneous and reactive singing. Where data were not normally distributed, we used paired samples Wilcoxon tests. Similarly, we used linear regressions to examine the relationship between inter-syllable frequency ratio and gap duration and Spearman’s rank correlation tests to check for relationships between inter-syllable frequency ratio and syllable duration.

## Additional files

Additional file 1:
**Spectrograms of subsequent syllables produced by a male skylark in response to a territorial playback and illustration of measures taken to apply the traditional upper-bound regression method.** (A, B) Examples with small gaps. (C, D) Examples with large gaps. Red stars indicate peak frequency of the end of the first syllable, and the start frequency of the subsequent syllable, the horizontal bar indicates gap duration, the vertical bar indicates inter-syllable frequency shift that was assessed by calculating the absolute difference of the end peak frequency of a given syllable type and the start peak frequency of the subsequent syllable. Additional file 2:
**Inter-syllable frequency shift plotted against gap duration using the traditional upper-bound regression method.** Black dotted line: upper-bound regression line. Red diamonds: syllables produced in response to territorial playbacks. Black dots: syllables produced in spontaneous song. Red diamonds and black dots belonging to the same bin of gap duration are displayed slightly out of alignment for better visibility. Black crosses: data points (assessed by applying equally sized bins [40]) used to calculate the upper-bound regression line). We binned measurements of gap duration on spectrograms into 2.9 ms increments - corresponding to the resolution of the FFT used for these measurements (FFT length 512; frame 100%; overlap 75%, Hamming window). The bin of gap durations with the highest inter-syllable frequency shift for the whole dataset was the 52 ms bin. As there should be no performance limit at gaps larger than this bin we considered all bins of gap durations less than or equal to 52 ms for the regression analysis but ignored bins of larger gap durations. We selected the maximum inter-syllable frequency shift for each subsequent 2.9 ms bin of gap duration (less than or equal to 52 ms) and calculated a linear regression through these maximum values (black crosses). Vocal gap deviation was measured as the minimum orthogonal distance of each data point to the upper-bound regression line; an example is shown for one data point (blue dotted line). Minimum area convex polygon is given in red for reactive and in black for spontaneous singing. Note that for smaller bins of gap durations (less than or equal to 52 ms) many data points lay on top of each other. The upper-bound regression has a significant positive slope (y = 85.455x + 629.68, linear regression: F_1,13_ = 44.86, r^2^ = 0.78, *P* = 0.00001). Additional file 3:
**Using the traditional upper-bound regression method revealed that vocal gap deviation was smaller when skylarks were singing in response to a territorial playback.** This indicates that they were singing closer to their performance limit when challenged than when singing spontaneously. Average values of all syllable types are shown for each of 16 subjects. Spontaneous singing: mean ± SD 41.53 ± 4.98; reactive singing: 38.85 ± 3.64; paired t-test, t = -2.56, df = 15, *P* = 0.02.

## Abbreviations

df: degrees of freedom
FFT: Fast Fourier transformation
SD: standard deviation
