# Supplementary figures and images for: Assessing vocal performance in complex birdsong: a novel approach
Source: BMC Biol. 2014 Aug 6;12:58. doi: 10.1186/s12915-014-0058-4 (PMC4243327; doi:10.1186/s12915-014-0058-4)

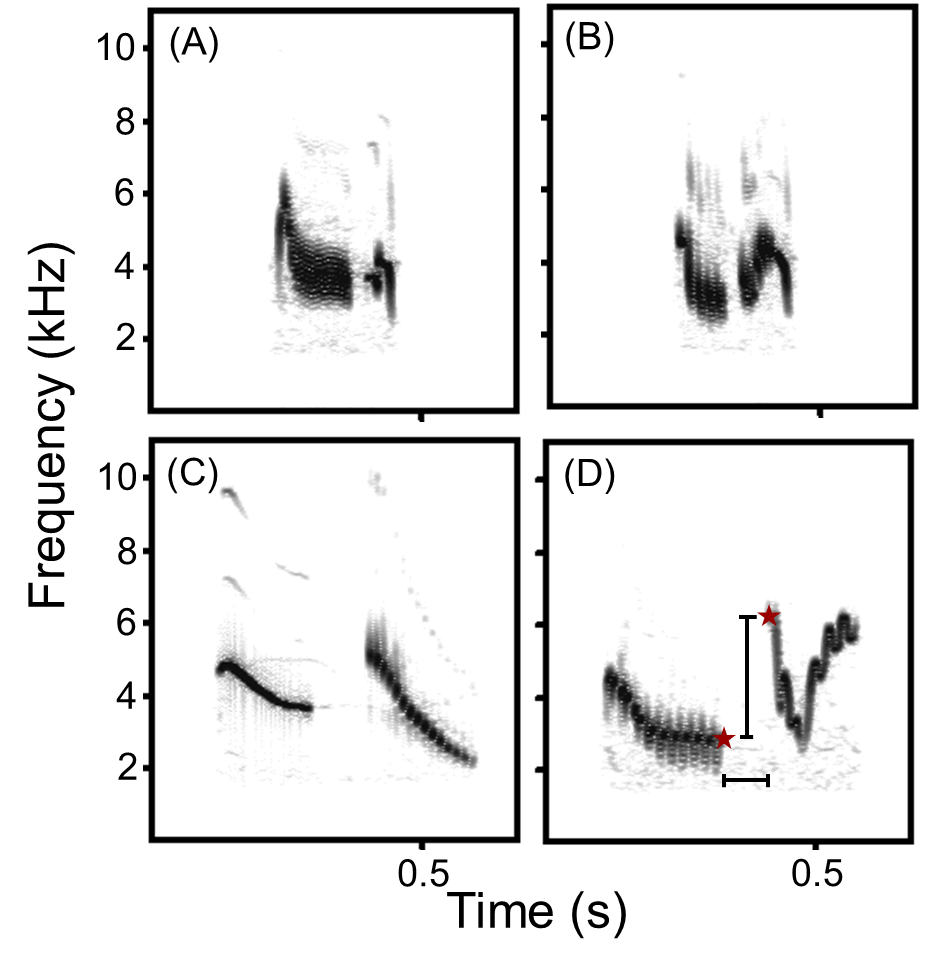

Supplement: Additional file 1: — Spectrograms of subsequent syllables produced by a male skylark in response to a territorial playback and illustration of measures taken to apply the traditional upper-bound regression method. (A, B) Examples with small gaps. (C, D) Examples with large gaps. Red stars indicate peak frequency of the end of the first syllable, and the start frequency of the subsequent syllable, the horizontal bar indicates gap duration, the vertical bar indicates inter-syllable frequency shift that was assessed by calculating the absolute difference of the end peak frequency of a given syllable type and the start peak frequency of the subsequent syllable. [file 12915_2014_58_MOESM1_ESM.zip › 1426797846122010_add1.bmp]

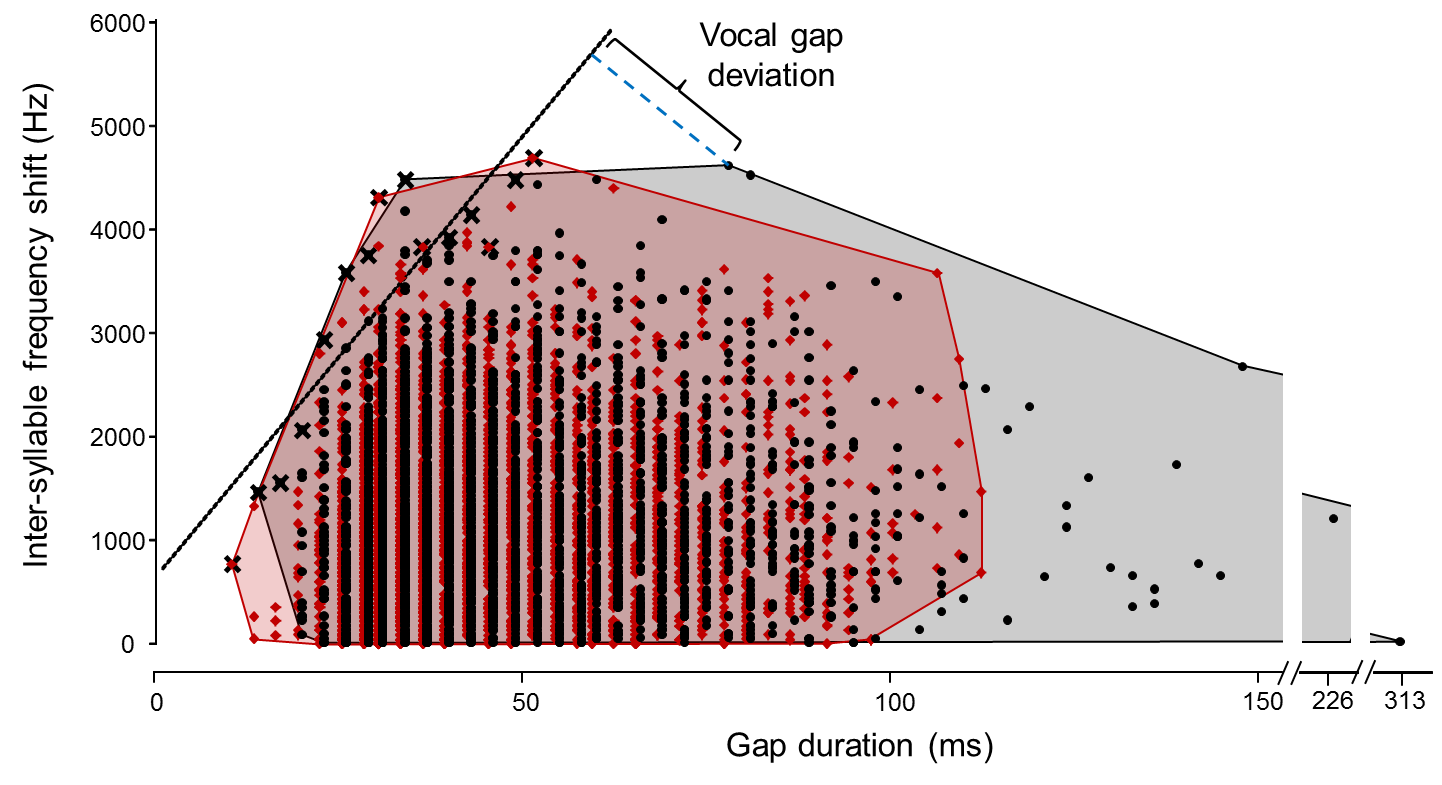

Supplement: Additional file 2: — Inter-syllable frequency shift plotted against gap duration using the traditional upper-bound regression method. Black dotted line: upper-bound regression line. Red diamonds: syllables produced in response to territorial playbacks. Black dots: syllables produced in spontaneous song. Red diamonds and black dots belonging to the same bin of gap duration are displayed slightly out of alignment for better visibility. Black crosses: data points (assessed by applying equally sized bins [40]) used to calculate the upper-bound regression line). We binned measurements of gap duration on spectrograms into 2.9 ms increments - corresponding to the resolution of the FFT used for these measurements (FFT length 512; frame 100%; overlap 75%, Hamming window). The bin of gap durations with the highest inter-syllable frequency shift for the whole dataset was the 52 ms bin. As there should be no performance limit at gaps larger than this bin we considered all bins of gap durations less than or equal to 52 ms for the regression analysis but ignored bins of larger gap durations. We selected the maximum inter-syllable frequency shift for each subsequent 2.9 ms bin of gap duration (less than or equal to 52 ms) and calculated a linear regression through these maximum values (black crosses). Vocal gap deviation was measured as the minimum orthogonal distance of each data point to the upper-bound regression line; an example is shown for one data point (blue dotted line). Minimum area convex polygon is given in red for reactive and in black for spontaneous singing. Note that for smaller bins of gap durations (less than or equal to 52 ms) many data points lay on top of each other. The upper-bound regression has a significant positive slope (y = 85.455x + 629.68, linear regression: F1,13 = 44.86, r2 = 0.78, P = 0.00001). [file 12915_2014_58_MOESM2_ESM.zip › 1426797846122010_add2.bmp]

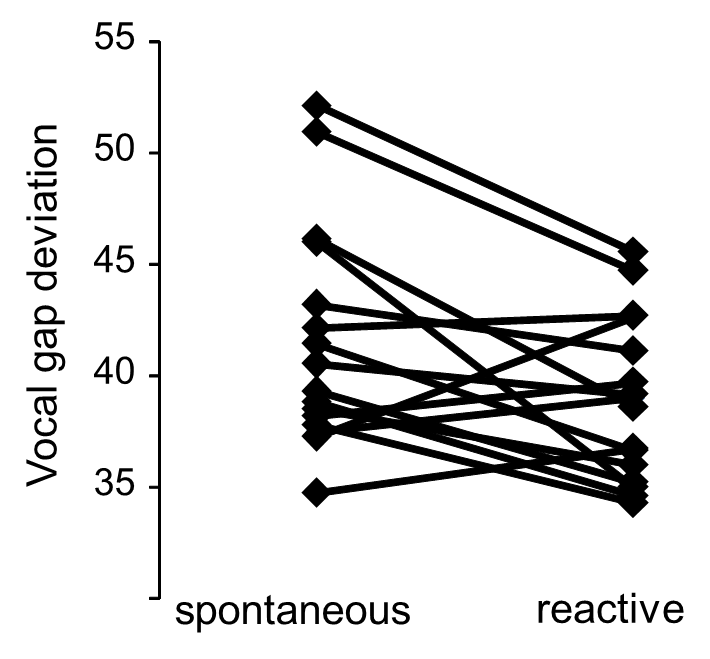

Supplement: Additional file 3: — Using the traditional upper-bound regression method revealed that vocal gap deviation was smaller when skylarks were singing in response to a territorial playback. This indicates that they were singing closer to their performance limit when challenged than when singing spontaneously. Average values of all syllable types are shown for each of 16 subjects. Spontaneous singing: mean ± SD 41.53 ± 4.98; reactive singing: 38.85 ± 3.64; paired t-test, t = -2.56, df = 15, P = 0.02. [file 12915_2014_58_MOESM3_ESM.zip › 1426797846122010_add3.bmp]
